# The impact of internet usage on the mental health of older adults in China: empirical analysis from a multi-dimensional perspective

**DOI:** 10.3389/fpubh.2024.1460558

**Published:** 2024-09-13

**Authors:** Wanjun Lu

**Affiliations:** College of Public Administration, China Resources & Environment and Development Academy (REDA), Nanjing Agricultural University, Nanjing, China

**Keywords:** internet usage, mobile payment, WeChat, depression index, China

## Abstract

**Background:**

The process of population aging in China is currently undergoing rapid acceleration. Simultaneously, the swift advancement of digitalization is fundamentally transforming individuals’ lifestyles. The usage of the internet and mobile internet tools by the older adults population is relatively inadequate. The issue of digital exclusion and its impact on the life quality of the older adult population has received significant attention.

**Objective:**

This study utilized microdata from the China Health and Retirement Longitudinal Study (CHARLS 2020) to empirically investigate the impact of internet usage on the mental health of older adult individuals. The depression index was utilized to assess the mental health, while four variables were employed to evaluate internet usage among the older adults in this study.

**Methods:**

The Center for Epidemiological Studies Depression Scale (CES-D) in CHARLS data was used to measure the depression index of older adults. Four variables including usage of internet, usage of WeChat, usage of WeChat moments and usage of mobile payment were used to represent the internet use of older adults, and there was progressive relationship between these four variables. In the empirical study, multiple regression analysis was adopted to empirically analysis the impact of internet usage on the mental health of older adults. In order to reduce the influence of endogenous problems on regression results, the propensity score matching method was used to verify the validity and robustness of regression results.

**Results:**

(1) Internet usage can significantly reduce the psychological depression of older adults and promote the formation of positive psychology; (2) With the increase of the depth of internet usage, especially the use of mobile internet and mobile payment, the internet use will have greater improvement effect on the depression; (3) The heterogeneity test found that there were certain differences in the impact of internet usage on different older adult groups; (4) Through a step-by-step analysis of 10 sub-indicators of depression index, the study found that Internet use mainly affected four indicators: “life hope,” “happiness,” “loneliness” and “life confidence,” while demonstrating no significant effect on other sub-indicators.

**Conclusion:**

According to the research, internet usage can significantly reduce the psychological depression index of the older adults and promote the formation of positive psychology. In China, digital exclusion is more prevalent in rural areas and among the less educated older adults. Public policies can be formulated to enhance internet adoption among these older adults population.

## 1 Introduction

China is experiencing rapid aging process. The number of people over 65 reached 217 million by the end of 2023, accounting for 15.4 percent of the total population (1). The aging process in China is anticipated to accelerate as the cohort born during the baby boom in the 1960s and 1970s gradually enters old age. Research shows that by 2050, the number of older adults over the age of 65 in China will reach 480 million, accounting for more than 34% of the total population (2). The World Health Organization advocates for active aging through the implementation of public policies, in response to the escalating global phenomenon of population aging (3). According to the advocacy of active aging, positive mental state is an important dimension. Insufficient social capital and limited social communication among older adults can significantly contribute to negative psychological conditions (4, 5), thereby presenting challenges in achieving goals related to active and healthy aging. Concurrently, China is currently undergoing a rapid process of digital transformation, which is fundamentally reshaping the paradigms of communication, employment, and financial transactions. The usage of internet can not only change the convenience of people’s lives, but also broaden social communication, which may have positive impact on the life quality (6, 7). Compared to developed countries, the educational attainment of the older adult population in China tends to be relatively low (8), the use of new technologies and intelligent device may be insufficient. According to the 53rd China Internet Development Statistics Report in 2023, the number of non-internet users in China is 317 million. Among them, people over 60 years old account for 39.8% (9). A substantial proportion of the older adult population faces the risk of digital exclusion, which may significantly impact their life quality.

Theoretically, the use of internet and mobile internet tools can facilitate the life of the older adults, such as online shopping, taxi hailing, etc. Simultaneously, mobile internet can effectively facilitate interpersonal communication, as well as providing them with more efficient access to external information. This may effectively alleviate feelings of loneliness among the older adult population and have positive impact on their psychological state. In the era of digitalization, internet usage includes the use of the internet to retrieve information, interpersonal communication, payment activities etc. In order to empirically analyze the impact of internet usage on the mental health of older adults in China, this study uses the data of the China Health and Retirement Longitudinal Study (CHARLS 2020). Based on statistical data and a comprehensive analysis of internet usage among the older adults, this study employs multiple linear regression and propensity score matching (PSM) methods to empirically examine the influence of internet usage across different latitudes on the mental health of older adults.

## 2 Materials and methods

### 2.1 Literature review

With the development of digital technology, using the internet and mobile phone is not just about getting more external information. Digital inclusion or exclusion will have significant impact on people’s lives (10). According to the socialization theory (11), individuals are encouraged to continuously acquire new skills in order to enhance their social integration and adaptation, thereby positively influencing their quality of life. Adapting to social changes and acquiring new skills can have important positive effects on the mental health of older people (12). Existing studies about the impact of internet usage on the health of the older adults are mainly conducted from the perspective of social capital and re-socialization theory. The research from the perspective of social capital suggests that internet usage can effectively enhance the extent and diversity of social communication among older adults, thereby exerting a positive impact on their individual lives. The theory of re-socialization posits that in an era characterized by perpetual change, the older adults adaptation to technological advancements and lifestyle transformations will yield a positive impact on their overall quality of life. According to the research based on the social capital theory, the use of internet can effectively expand the range of social interaction of older adults, reduce the loneliness and other negative emotions, and improve psychological state (13, 14). Some studies suggest that internet use may also lead to lower community participation and community activities among the older adults. This will have negative impact on subjective well-being (15, 16). The ultimate impact of internet usage on the life quality depends on whether it has positive effects on social capital.

Existing studies generally use certain latitude to indicate the mental health status of the older adults. Some studies believe that life satisfaction is highly correlated with mental health and subjective well-being (17, 18). Using the RCT method, the study found that providing internet training to older adults effectively increased their self-rated life satisfaction (19). If the usage of internet is differentiated, only the usage of internet for entertainment and leisure can effectively improve life satisfaction of older adults (20, 21). Some studies based on psychological status have found that the usage of internet can effectively reduce loneliness of older adults (22, 23). Other studies have found that older adults who use the internet generally have lower level of depression and higher level of subjective well-being (24–26). The probability of digital exclusion differs across various older adults demographics. The older people with higher levels of education are more inclined to use internet, and are also more likely to experience positive psychological impacts from the usage (27). In addition, the usage of internet by the older adults is significantly influenced by factors such as social status, economic level, and living arrangements (28, 29). In addition, some studies found that the internet can facilitate older adult individuals’ acquisition of relevant knowledge for promoting health management and physical well-being (30–32). A cross-country comparative study revealed a more pronounced issue of digital exclusion among the older adults in China. The phenomenon of digital exclusion can have detrimental effects on cognitive level of older adults, including focus, memory, and executive function (33).

In China, mobile payment is more convenient and popular. WeChat has become an important and timely communication tool for interpersonal communication. Based on existing researches, this study will consider internet use from a more detailed perspective, including the utilization of the internet, mobile communication software, and mobile payment, in order to comprehensively reflect the potential impact of internet usage on the mental health of older adult individuals. The study will also explore the internal heterogeneity within the older adult population and investigate the potential impact of internet usage on different subgroups of older individuals.

### 2.2 Data sources

The dataset utilized in this research is the China Health and Retirement Longitudinal Survey (CHARLS 2020). The data was designed to track individuals aged 45 years and above. In this dataset, the information about the family, work, life, and community characteristics were collected. The data is currently the most representative micro-data for studying the health status and living conditions of older adults in China, and the demographic characteristics of the baseline CHARLS sample are very similar to those found in the results of the 2010 census (34). The 2020 dataset comprised a total of 19,395 samples, with 10,654 samples specifically collected from the older adult population aged over 60. A series of important indicators such as internet use, physical condition, depression index among the older adults were collected, which can meet the research needs.

### 2.3 Variables

#### 2.3.1 Dependent variables

The study used depression index to indicate mental health of older adults. Some studies have shown that mental health is highly correlated with life quality, depression index is also a comprehensive indicator of individual mental health status (35, 36). In the CHARLS data, the psychological depression state of individuals was assessed using a set of 10 questions based on the CES-D scale. This scale can largely reflect individual psychological depression level, and has been widely used in related researches (37, 38). The answers to each question included “rarely or not,” “not much,” “sometimes or half of the time” and “most of the time.” The negative questions were assigned scores of 0, 1, 2, and 3 for the four response options in this study. Conversely, the forward questions were scored with values of 3, 2, 1, and 0 corresponding to the four response directions. The overall score of the depression index ranges from 0 to 30, with higher scores indicating more severer of depression.

#### 2.3.2 Independent variables

Internet usage is the core independent variable of the study. With the rapid advancement of digitalization, the role and function of the internet have undergone significant transformations. Relevant studies primarily investigate the internet usage among the older adult population, employing a singular variable to assess the usage of internet (39). With the development of mobile internet technology, the communication, information sharing and online payment of mobile internet are having wider impact on people’s lives. In China, platforms such as WeChat and the associated features like WeChat Moments, mobile payment systems have brought about substantial changes in people’s daily lives. Multiple indicators are used in this study to represent older adults’ utilization of the internet. The internet-use variable is derived from the question in the questionnaire: “Have you utilized the internet within the previous month?” The response of 1 indicates affirmative usage, while a response of 0 signifies no usage. WeChat is becoming a widely used communication APP in China. According to data from Tencent’s annual report, WeChat accounts in China have exceed 1.3 billion in 2023. The variable “chat” is derived from the question in the questionnaire, which asks: “Do you use WeChat?” The answer is assigned 1 for usage and 0 for non-usage. The variable “pay” is derived from the question in the questionnaire: “Will you pay with mobile phone?” The value of 1 is assigned to a positive answer, while the value of 0 is assigned to a negative answer. In China, WeChat Moments have an important function for private sharing and can be used as a proxy variable for internet usage proficiency. The variable “chat_m” is generated based on the question in the questionnaire: “Do you post on webchat moments?” with a value of 1 for “yes” and 0 for “no”.

#### 2.3.3 Control variables

Control variables are determined based on relevant studies in the academic literature. The control variables will influence individuals’ internet usage, and also impact on the life quality and mental health. The control variables include: age, education, disability level, personal income, place of residence, spouse, work, number of children, and live with children or not. The study transformed education into a continuous variable according to the education level answered by the respondents, illiteracy is assigned value 0, no primary school graduation is assigned value 1, primary school is assigned value 6, junior high school is assigned value 9, senior high school is assigned value 12, technical secondary school is assigned value 13, junior college is assigned value 15, and bachelor degree or above is assigned value 16. According to the respondent’s reported place of residence, urban is assigned value 1 and rural is assigned value 0. The study utilized the information obtained from the questionnaire to classify individuals into two groups: those with a spouse (coded as 1) and those without a spouse (coded as 0), generating the variable “spouse.” The variable “personal income” is derived by aggregating the itemized income levels provided by the respondents.

This study evaluated the disability status of the older adults according to the subjective report of activity of daily living (ADL) and instrumental activity of daily living (IADL). The ADL index includes six items: eating, dressing, going to the toilet, getting in and out of bed, taking a bath and moving around indoors. The IADL index includes household activities: cooking, shopping, remembering phone numbers, remembering time to take medicine, managing finances. The ADL index contains the basic requirements for independent living, and failure to meet one may directly lead to loss of self-care (40, 41). IADL index is mainly related to personal life quality (42). Answers to any of the six questions related to the ADL index as “difficulty, need help” or “inability to complete” defined the older adults as severely disabled. If all six questions in the ADL index can be completed, but one of the six questions in the IADL index is answered as “difficulty, need help” or “cannot be completed,” it is defined as moderate disability. Since the answers to the 12 questions in designing ADL and IADL are: “No difficulty,” “difficulty but can still be completed,” “difficulty, need help,” and “cannot be completed,” the four questions are assigned values 1, 2, 3, and 4, respectively. ADL and IADL will be formed by summing up, with a value of 6–24, with higher scores indicating more severe disability.

#### 2.3.4 Variable descriptions and descriptive statistics

The study focused on individuals aged 60 to 85. The minimum age for the older adult population is set at 60, while previous studies have established an upper age limit of either 80 or 85 years. Within the CHARLS data, a higher proportion of family members responded on behalf of individuals aged over 85, and the vacancy of depression scale was more obvious. However, extending the upper age limit for this study may introduce additional challenges in sample selection. Furthermore, age was utilized as a control variable to further mitigate the impact of age differences on regression results. Table 1 presents the descriptive statistical results of the variables. The results of the descriptive statistics indicate that the internet usage rate among older adults is 21.8%. In the era of mobile internet, 18.5% of older adult individuals can utilize WeChat as a communication tool. 10.5% of older adult individuals are proficient in sharing WeChat moments with their friends. Mobile payment adoption is relatively low among older adults, with only 9.8% being able to utilize mobile payment tools effectively. According to the descriptive statistics, it can be inferred that digital exclusion may be common in older adults, especially the proportion of older adult individuals proficient in using mobile internet tools is relatively low. The potential correlation could be attributed to the educational attainment of the older adult population. It is worth noting that China initiated 9-year compulsory education from 1986 (43). The individuals included in the sample were born prior to this period, resulting in a generally low level of education with an average duration of 4.2 years. Within the sample, approximately 9.3% of older adults exhibit severe disability, while around 15.2% have moderate disability levels, respectively. Overall, older adults demonstrate lower depression levels with an average score of 9.42 on the depression index.

**Table 1:** Reports the variable descriptions and descriptive statistics results.

category	Variable name	Symbol	Mean	S.D.	Min	Max	*N*
Dependent variable	Depression	Dep	9.420	6.399	0	30	9,329
Independent variables	Internet-use	Inter	0.218	0.413	0	1	10,373
	Wechat usage	Chat	0.185	0.388	0	1	10,373
	Wechat moment	chat_m	0.105	0.307	0	1	10,379
	Mobile payment	Pay	0.098	0.297	0	1	10,373
Control variable	Age	Age	68.503	6.219	60	85	10,393
	education	Edu	4.217	4.430	0	16	10,393
	Gender	Gender	0.485	0.500	0	1	10,393
	Have a spouse	Spou	0.775	0.417	0	1	10,393
	ADL level	ADL	6.962	2.452	6	24	10,373
	IADL level	IADL	7.719	3.684	6	24	10,371
	Severe disability	s_dis	0.093	0.290	0	1	10,379
	Moderate disability	m_dis	0.152	0.359	0	1	10,379
	Live with child	Live	0.321	0.467	0	1	10,390
	Number of children	Child	2.759	1.215	0	5	10,390
	Income	In	11,480	19,979	0	568,430	10,372
	Pension	Pen	9,338	17,981	0	567,200	10,372
	Work	Work	0.524	0.499	0	1	10,374
	Resident (urban/rural)	Res	0.236	0.425	0	1	10,381

### 2.4 Methods

The study used multiple linear regression to analyze the effects of different latitudes of internet use on mental health. Since there is a progressive relationship among the four variables explained, collinearity will occur when all four variables are included in the equation simultaneously. The study used four independent regression equations to analyze the effects of different degrees and latitudes of Internet use on the mental health of older adults. The Equations 1–4 are the regression equations, where X_i_ denote the control variables, and the research focuses on examining the significance of coefficients $\beta_{1}$~$\beta_{4}$. The difference in regression coefficients $\beta_{1}$~$\beta_{4}$ can simultaneously indicate the extent of improvement in mental health status among older adult individuals as the level of internet usage increases.


(1) $$\text{depress}=\alpha_{1}+\beta_{1}\cdot \text{inter}+\sum \gamma_{i}\cdot X_{i}+\varepsilon$$

(2) $$\text{depress}=\alpha_{2}+\beta_{2}\cdot \text{chat}+\sum \gamma_{i}\cdot X_{i}+\varepsilon$$

(3) $$\text{depress}=\alpha_{3}+\beta_{3}\cdot \text{chat}\_m+\sum \gamma_{i}\cdot X_{i}+\varepsilon$$

(4) $$\text{depress}=\alpha_{4}+\beta_{4}\cdot \text{pay}+\sum \gamma_{i}\cdot X_{i}+\varepsilon$$

During the empirical study, participants were categorized based on their disability status, level of education, and place of residence. The heterogeneity of the impact of internet usage on different groups was analyzed. After considering the influence of endogeneity on regression results, propensity score matching (PSM) was employed to verify the robustness and validity of the conclusions. Finally, an empirical study will be conducted to regress the sub-indexes of depression separately in order to test the specific influencing mechanism.

## 3 Empirical study

### 3.1 Regression results

The four independent variables can represent the proficiency and depth of the usage of Internet. Among them, the internet usage is the most extensive, followed by the use of WeChat, followed by the use of WeChat moments, and finally the use of mobile payment. Given the progressive relationship among the four indicators, it is imperative to conduct separate regression analyses in order to present the findings effectively. Firstly, the study adopts multiple linear regression to measure the influence of different degrees of internet usage on the depression index of older adults. The regression results are shown in Table 2. Regression results show that internet usage can significantly reduce the degree of depression in the older adults. The regression results of the four dependent variables indicate that with increasing depth of internet usage, that the internet has a more significant impact on reducing depression levels among the older adults. The exposure and usage of the internet can reduce the depression index of older adults by 0.521 (*p* < 0.01). If the older adults used WeChat, internet usage could reduce the depression index of older adults by 0.565 (*p* < 0.01). The utilization of WeChat moments sharing function among the older adults is associated with a significant reduction in their depression index by 0.837 (*p* < 0.01). If the older adults were able to use mobile payment, the overall depression index decreased to 1.158 (*p* < 0.01). Since the average depression index score of the older adults is only 9.42, it can be argued that internet usage plays a pivotal role in ameliorating the depressive symptoms among the older adults. There may be selective bias in the effects of internet usage on the mental health of older adults. Among the control variables, income can significantly reduce the degree of depression, but it is not shown in the table because the coefficient is too small. Physical health is an important control variable, and studies controlling for ADL and IADL indexes show that disability does significantly increase depression level in the older adults.

**Table 2:** The impact of internet utilization on the depression index.

	Depression			
	(1)	(2)	(3)	(4)
Inter	−0.521^***^ (0.167)			
Chat		−0.565^***^ (0.174)		
chat_m			−0.837^***^ (0.203)	
Pay				−1.158^***^ (0.204)
Age	−0.047^***^ (0.013)	−0.047^***^ (−0.013)	−0.044^***^ (0.012)	−0.048^***^ (0.012)
Edu	−0.064^***^ (0.017)	−0.064^***^ (0.017)	−0.063^***^ (0.017)	−0.057^***^ (0.017)
Gender	−1.187^***^ (0.134)	−1.193^***^ (0.134)	−1.200*** (0.134)	−1.178^***^ (0.133)
Spou	−1.144^***^ (0.170)	−1.147^***^ (0.170)	−1.140^***^ (0.170)	−1.152^***^ (0.170)
ADL	0.631^***^ (−0.059)	0.630^***^ (−0.059)	0.631^***^ (−0.059)	0.630^***^ (−0.059)
IADL	0.311^***^ (0.034)	0.311^***^ (−0.034)	0.311^***^ (−0.034)	0.310^***^ (−0.034)
Live	−0.339^**^ (0.134)	−0.342^**^ (0.134)	−0.342^**^ (0.134)	−0.352^***^ (0.134)
Child	0.083 (0.060)	0.082 (0.060)	0.073 (0.060)	0.078 (0.060)
Work	0.499^***^ (0.141)	0.497^***^ (0.141)	0.493^***^ (0.141)	0.493^***^ (0.141)
Res	−0.468^**^ (0.186)	−0.471^**^ (0.186)	−0.463^**^ (0.184)	−0.457^**^ (0.183)
In	Yes	Yes	Yes	Yes
Cons	8.135^***^ (0.935)	8.115^***^ (0.931)	8.213^***^ (0.920)	7.960^***^ (0.916)
*N*	9,321	9,321	9,321	9,321
Adjust R^2^	0.156	0.156	0.156	0.157

Robust standard errors in parentheses. ^***^p < 0.01, ^**^p < 0.05, ^*^p < 0.1.

### 3.2 Heterogeneity test

There may be significant inter-group heterogeneity in this research. To test for inter-group heterogeneity and obtain more robust regression results, the study analysis the heterogeneity from three dimensions: disability, education and residence. Table 3 shows the results of the heterogeneity analysis based on disability. Disability is more likely to result in more severe mental health issues (44, 45). The study included moderately and severely disabled older adults as one group, and others as another group. The results showed that internet usage and WeChat did not have significant positive impact on the depression index of disabled older adults. However, as the older adults learn to use mobile payment tools, it can significantly improve reduce their depression index by 1.540 (*p* < 0.05). The access to information and interpersonal communication may not be the primary concerns for older adult individuals with disabilities. Consequently, the mere exposure and utilization of internet do not necessarily yield a positive impact on their psychological well-being. However, when internet usage significantly enhances their life quality, it can have a favorable effect on their mental health. The convenience provided by online payments enables disabled older adult individuals to easily acquire services and goods, thereby positively influencing their mental well-being. Regression results of non-disabled older adults showed that all dimensions of internet usage could significantly reduce the depression level. Compared to the disabled older adults, the impact of mobile payment on the psychological depression index of non-disabled individuals is relatively lower, potentially due to their ability to utilize offline payment methods.

**Table 3:** Heterogeneous analysis of disability.

	Depress							
	Non-disabled older adults				Disabled older adults			
	(1)	(2)	(3)	(4)	(5)	(6)	(7)	(8)
Inter	−0.606^***^ (0.171)				0.106 (0.467)			
Chat		−0.611^***^ (0.176)				0.060 (0.504)		
chat_m			−0.732^***^ (0.202)				−0.998 (0.713)	
Pay				−0.988^***^ (0.203)				−1.540^**^ (0.773)
Control variables	Yes	Yes	Yes	Yes	Yes	Yes	Yes	Yes
Cons	−4.998^***^ (1.326)	−5.038^***^ (1.325)	−5.341^***^ (1.310)	−5.032^***^ (1.314)	14.4^***^ (1.752)	14.6^***^ (1.750)	15.0^***^ (1.703)	15.4^***^ (1.733)
*N*	7,351	7,351	7,351	7,351	2,201	2,201	2,201	2,201
Adjust R^2^	0.148	0.148	0.148	0.149	0.083	0.083	0.083	0.083

Robust standard errors in parentheses. ^***^p < 0.01, ^**^p < 0.05, ^*^p < 0.1. The control variables are consistent with Table 1.

In CHARLS 2020 data, 25.4% of the older adults aged 60–85 have received junior high school education or above, and only 10.2% have received senior high school education or above. The insufficient education presents a substantial obstacle to the utilization of the internet among older individuals (46). The study further examined the heterogeneity of education groups, which were categorized into two subgroups based on their attainment of junior high school education. The main results are reported in Table 4. The results revealed that neither the use of the internet nor WeChat alone reduced depression index among individuals with primary education or below. However, as the older adults skillfully use the WeChat Moments function, the internet usage will significantly reduce the degree of depression in the older adults. The utilization of mobile payment has a more favorable impact on older adult individuals with lower educational attainment, resulting in an average reduction of their depression index by 1.281 (*p* < 0.01). In the group with secondary education and above, internet usage at different latitudes can significantly reduce the degree of depression. Compared with lower education group, mobile payment has less impact on the psychological depression index. The reason for the difference in the results may be due to the fact that the older adults with lower education may have reading difficulties in using the internet. However, the use of WeChat sharing function can expand their social interaction, and mobile payment can significantly improve the convenience of life, which will have a positive impact on individual mental health. For the older adults with higher education level, they can experience the convenience of reading and information acquisition brought by the internet, and improve their mental health.

**Table 4:** Heterogeneous analysis of education.

	Depress							
	Primary school and below				Junior high school and above			
	(1)	(2)	(3)	(4)	(5)	(6)	(7)	(8)
Inter	−0.222 (0.226)				0.659^***^ (0.247)			
Chat		−0.196 (0.243)				−0.700^***^ (0.247)		
chat_m			−0.883^***^ (0.328)				−0.535^***^ (0.258)	
Pay				−1.281^***^ (0.345)				−0.756^***^ (0.254)
Control variables	Yes	Yes	Yes	Yes	Yes	Yes	Yes	Yes
Cons	9.413^***^ (1.095)	9.351^***^ (1.090)	9.502^***^ (1.077)	9.579^***^ (1.076)	5.196^***^ (1.841)	5.304^***^ (1.840)	4.589^***^ (1.793)	5.088^***^ (1.818)
*N*	6,878	6,878	6,878	6,878	2,443	2,443	2,443	2,443
Adjust R^2^	0.118	0.118	0.118	0.119	0.190	0.190	0.189	0.190

Robust standard errors in parentheses. ^***^p < 0.01, ^**^p < 0.05, ^*^p < 0.1. The control variables are consistent with Table 1.

There is a huge gap between urban and rural areas in China (47), and this gap is reflected in many dimensions such as income level, education and health status (48). According to CHARLS2020 data, only 13.7% of the older adults aged 60–85 in rural areas use the internet, while the proportion of urban older adults using the internet is about 43.9%. The comparison suggests that digital exclusion faced by older adult individuals in rural areas may be more apparent. The study examined inter-group heterogeneity using urban and rural subgroups of residence. The main regression results are presented in Table 5. Group test showed that mobile payment in rural areas could significantly reduce the depression index of older adults by 1.281 (*p* < 0.01). However, the impact of the internet usage on depression index among older adult individuals in rural areas, however, demonstrated a relatively low significance (*p* < 0.1). This may be attributed to the limited educational attainment among older adult individuals residing in rural areas. In urban areas, the different latitude of internet usage has positive effects on the psychological depression of older adults. However, compared with rural areas, the degree of influence of mobile payment on urban older adults is lower (1.012, *p* < 0.01).

**Table 5:** Heterogeneous analysis of resident.

	Depress							
	Rural				Urban			
	(1)	(2)	(3)	(4)	(5)	(6)	(7)	(8)
Inter	−0.738^*^ (0.212)				−0.737^***^ (0.276)			
Chat		−0.537^**^ (0.226)				−0.551^**^ (0.274)		
chat_m			−0.863^***^ (0.303)				−0.830^***^ (0.273)	
Pay				−1.281^***^ (0.302)				−1.012^***^ (0.280)
Control variables	Yes	Yes	Yes	Yes	Yes	Yes	Yes	Yes
Cons	8.798^***^ (1.071)	8.876^***^ (1.067)	8.758^***^ (1.054)	8.970^***^ (1.056)	6.109^***^ (1.919)	5.739^***^ (1.913)	5.624^***^ (1.858)	5.968^***^ (1.879)
*N*	7,176	7,176	7,176	7,176	2,145	2,145	2,145	2,145
Adjust R^2^	0.127	0.127	0.127	0.128	0.183	0.183	0.184	0.185

Robust standard errors in parentheses. ^***^p < 0.01, ^**^p < 0.05, ^*^p < 0.1. The control variables are consistent with Table 1 except the variable “resident”.

### 3.3 Robust test

The heterogeneity analysis was conducted in various ways, and the results of the heterogeneity regression were generally consistent with theoretical expectations. However, biased estimates may still arise due to selective bias and other endogenous issues. To mitigate the impact of endogeneity problems on the regression analysis results, propensity score matching method was employed for further verification. The study first matched the main covariates designed in the regression analysis of the study, and calculated the average treatment effect of the treatment group (ATT) after satisfying the propensity score test. The treatment effect is calculated using three methods: near matching, nuclear matching, and Mahalanobis distance. There is no systematic difference in the results of multiple matching methods. The results obtained from 1:1 no-put-back proximity matches are presented in Table 6. The results of propensity score matching showed that after taking into account the sample balance, different dimensions of internet usage can significantly improve the degree of psychological depression in the older adults. The results show the validity and robustness of the above analysis.

**Table 6:** Propensity score matched analysis results.

		Experimental group	Control group	ATT	*N*
(1)	Inter	7.646	8.451	−0.805^***^ (0.188)	9,321
(2)	Chat	7.452	8.281	−0.829^***^ (0.200)	9,321
(3)	chat_m	6.691	7.604	−0.913^***^ (0.252)	9,321
(4)	Pay	6.338	7.366	−1.028^***^ (0.251)	9,321

Standard error in parentheses. ^***^p < 0.01.

## 4 Further discussion

The depression index variable utilized in this study comprised ten dimensions, each latitude involves a question with four choices, assigning values of 1, 2, 3, and 4 to each response. It is plausible to further scrutinize the impact of internet usage on the depression index across various dimensions. The mental health impact and underlying mechanisms of internet usage on older adults can be further explored through a more comprehensive latitude analysis. The study conducted stepwise regression analysis and identified that the internet primarily influenced four dimensions: “life hope,” “happiness,” “loneliness,” and “life confidence.” No significant impact was observed on the remaining six dimensions. Table 7 presents the regression results specifically focusing on the significant effects of internet usage. The regression outcomes related to internet usage and mobile payment variables are provided. In order to be consistent with the variables in the depression index above. The three variables “life hope,” “happiness,” and “life confidence” were assigned inverse values in the study, with lower scores indicating better presence of positive psychological emotions. The results show that different latitudes of internet usage can significantly improve older people’s happiness and hope for future life. Simultaneously, the utilization of the internet can alleviate the loneliness experienced by older adult individuals. In addition, internet usage improves the confidence of the older adults about their future life. In all regression analyses, as the depth of internet usage increases, specifically from basic internet usage to acquiring mobile payment skills, the magnitude of the impact of Internet use on older adult individuals mental health also intensifies.

**Table 7:** Multi-latitude regression analysis results.

	Life hope		Happiness		Loneliness		CONFIDENCE	
	(1)	(2)	(3)	(4)	(5)	(6)	(7)	(8)
Inter	−0.210^***^ (0.036)		−0.235^***^ (0.039)		−0.057^**^ (0.026)		−0.069^***^ (0.024)	
Pay		−0.288^***^ (0.046)		−0.309^***^ (0.050)		−0.062^**^ (0.031)		−0.098^***^ (0.025)
Control variables	Yes	Yes	Yes	Yes	Yes	Yes	Yes	Yes
Cons	1.067^***^ (0.199)	0.992^***^ (0.196)	1.069^***^ (0.230)	0.978^***^ (0.207)	0.631^***^ (0.162)	0.600^***^ (0.159)	0.415^***^ (0.150)	0.391^***^ (0.148)
*N*	9,321	9,321	9,321	9,321	9,321	9,321	9,321	9,321
Adjust R^2^	0.053	0.053	0.065	0.065	0.115	0.115	0.115	0.115

Robust standard errors in parentheses. ^***^p < 0.01, ^**^p < 0.05, ^*^p < 0.1. The control variables are consistent with Table 1.

## 5 Conclusion and implications

The rapid advancement of digitization and information technology constitutes a pivotal characteristic of the contemporary era. Due to the rapid advancement in internet technology, the internet and mobile technologies have become indispensable tools for individuals to access information, social communication, and shopping. The utilization of internet technology theoretically enhances convenience in individuals’ lives, while simultaneously expanding the scope of their social interactions. The multifaceted avenues simultaneously promote the growth and nurturing of positive psychology. Older adult individuals exhibit a diminished aptitude for acquiring and adapting to novel technologies (49, 50). The older adult population in China generally possesses a limited educational background, which poses a significant obstacle to their internet utilization. Using micro data from the CHARLS2020 survey, this study investigated the influence of internet usage on the mental health of older adults, with depression index as an indicator for assessing their mental health. To comprehensively capture various dimensions of internet usage, particularly considering the utilization of mobile Internet tools, four variables were introduced to represent older adult individuals’ engagement with the Internet: internet usage, WeChat usage, WeChat moments usage, and mobile payment.

The main findings of the study include: (1) Internet usage can significantly reduce the psychological depression level of the older adults and promote positive psychology; (2) With the increase of the depth of internet usage, especially the use of mobile internet and mobile payment, the internet usage will have greater improvement effect on the degree of depression of older adults; (3) The heterogeneity test found that there were certain differences in the impact of internet use on different older adult groups. Among the disabled older adults, only the mobile payment function can significantly improve their psychological conditions, and the simple use of the internet and WeChat has no significant impact on their mental health. Based on the heterogeneity test of education level, it is found that the internet can have positive impact on the mental health of older adults with an education level below junior middle school only when they learn to use WeChat moments or mobile payment function. In order to mitigate the influence of endogenous issues, the propensity score matching method was employed to validate the robustness of the research findings. Simultaneously, a sub-analysis was conducted on the composition of the depression index, revealing that among its 10 sub-indicators, internet usage predominantly exhibited positive impact on “life hope,” “happiness,” “loneliness,” and “life confidence,” while demonstrating no significant effect on other indicators. According to the research, in the era of digitalization and aging, the digital inclusion of older adults can be promoted through corresponding policies, which may have positive impacts on the mental health and convenience of life of the older adults. Based on the research conclusions, digital exclusion remains prevalent among the older adult population in China. Implementing effective internet utilization services through community initiatives and volunteer efforts may enhance the likelihood of older adult individuals engaging with online platforms. Furthermore, efficient public services can effectively mitigate potential adverse impacts of digital exclusion on the mental well-being and overall quality of life experienced by older adults. Certainly, the excessive utilization of the internet can also give rise to digital addiction and dependency (51, 52), which will be the primary focus of forthcoming research.

## Data Availability

Publicly available datasets were analyzed in this study. This data can be found here: https://charls.charlsdata.com/pages/Data/2020-charls-wave5/zh-CN.html.
